# Bone marrow mesenchymal stem cells facilitate diabetic wound healing through the restoration of epidermal cell autophagy via the HIF-1α/TGF-β1/SMAD pathway

**DOI:** 10.1186/s13287-022-02996-9

**Published:** 2022-07-15

**Authors:** Yan Shi, Shang Wang, Weiwei Zhang, Yihan Zhu, Zhiqiang Fan, Yuesheng Huang, Furong Li, Ronghua Yang

**Affiliations:** 1 Translational Medicine Collaborative Innovation Center, Shenzhen People’s Hospital (The Second Clinical Medical College of Jinan University; The First Affiliated Hospital, Southern University of Science and Technology), Shenzhen, 518055 China; 2 Guangdong Engineering Technology Research Center of Stem Cell and Cell Therapy, Shenzhen Key Laboratory of Stem Cell Research and Clinical Transformation, Shenzhen, 518020 China; 3 The First Affiliated Hospital, Jinan University, Guangzhou, 510632 China; 4 Chongqing Key Laboratory of Traditional Chinese Medicine for Prevention and Cure of Metabolic Diseases, Chongqing Medical University, Chongqing, 400016 China; 5 Department of Plastic and Aesthetic Surgery, Jiangxi Maternal and Child Health Hospital, Nanchang, 330006 China; 6 Orthopaedic Trauma, Jiangxi Provincial People’s Hospital (The First Affiliated Hospital of Nanchang Medical College), Nanchang, 330006 Jiangxi China; 7 Department of Wound Repair, Institute of Wound Repair and Regeneration Medicine, Southern University of Science and Technology Hospital, Southern University of Science and Technology School of Medicine, Shenzhen, 518055 China; 8 Department of Burn and Plastic Surgery, Guangzhou First People’s Hospital, South China University of Technology, Guangzhou, 510180 Guangdong China

**Keywords:** Bone marrow mesenchymal stem cells, TGF-β1, Autophagy, Diabetic wound

## Abstract

**Background:**

The biological activity and regenerative medicine of bone marrow mesenchymal stem cells (BMSCs) have been focal topics in the broad fields of diabetic wound repair. However, the molecular mechanisms are still largely elusive for other cellular processes that are regulated during BMSC treatment. Our previous studies have shown that hypoxia is not only a typical pathological phenomenon of wounds but also exerts a vital regulatory effect on cellular bioactivity. In this study, the beneficial effects of hypoxic BMSCs on the cellular behaviors of epidermal cells and diabetic wound healing were investigated.

**Method:**

The viability and secretion ability of hypoxic BMSCs were detected. The autophagy, proliferation and migration of HaCaT cells cultured with hypoxic BMSCs-derived conditioned medium were assessed by estimating the expression of autophagy-related proteins, MTS, EdU proliferation and scratch assays. And the role of the SMAD signaling pathway during hypoxic BMSC-evoked HaCaT cell autophagy was explored through a series of in vitro gain- and loss-of-function experiments. Finally, the therapeutic effects of hypoxic BMSCs were evaluated using full-thickness cutaneous diabetic wound model.

**Results:**

First, we demonstrated that hypoxic conditions intensify HIF-1α-mediated TGF-β1 secretion by BMSCs. Then, the further data revealed that BMSC-derived TGF-β1 was responsible for the activation of epidermal cell autophagy, which contributed to the induction of epidermal cell proliferation and migration. Here, the SMAD signaling pathway was identified as downstream of BMSC-derived TGF-β1 to regulate HaCaT cell autophagy. Moreover, the administration of BMSCs to diabetic wounds increased epidermal autophagy and the rate of re-epithelialization, leading to accelerated healing, and these effects were significantly attenuated, accompanied by the downregulation of Smad2 phosphorylation levels due to TGF-β1 interference in BMSCs.

**Conclusion:**

In this report, we present evidence that uncovers a previously unidentified role of hypoxic BMSCs in regulating epidermal cell autophagy. The findings demonstrate that BMSC-based treatment by restoring epidermal cell autophagy could be an attractive therapeutic strategy for diabetic wounds and that the process is mediated by the HIF-1α/TGF-β1/SMAD pathway.

**Supplementary Information:**

The online version contains supplementary material available at 10.1186/s13287-022-02996-9.

## Background

Diabetic foot is one of the major complications of diabetes mellitus and leads to ulcerations and amputations of the lower extremities. Impaired self-healing capability is a common cause of diabetic foot ulcers and limb amputations. Although a growing number of biotechnology applications have been reported for diabetic wound healing and soft tissue defects [1–3], the refractory characteristic of diabetic wounds is still a major clinical problem that must be addressed. In normal skin, wounding induces epidermal cell autophagy to degrade misfolded or damaged proteins and organelles, which in turn provides essential elements for cell metabolism and survival, and this process has close connections to the wound repair process [1, 4]. Our previous study showed that epidermal cell autophagy was inhibited in diabetic skin, which subsequently compromised epidermal cell functions, including proliferation and emigration, as well as further re-epithelialization, leading to delayed healing or no healing [5, 6]. Thus, dysfunction of epidermal cell autophagy is considered to be a significant pathophysiological change in diabetic skin during wound healing, and it undoubtedly provides support to further improve defects in epidermal cell autophagy as a therapeutic strategy for diabetic wounds.

Bone marrow mesenchymal stem cells (BMSCs) are multipotent stem cells that have demonstrated the potency to self-renew and differentiate into multiple lineages. Compelling evidence has shown the therapeutic effects of BMSCs on tissue repair [7, 8]. Although BMSC-based therapies for wound healing have been introduced in clinical trials [9], the molecular mechanisms are still largely elusive for other cellular processes that are regulated during BMSC treatment and determine cell apoptosis, autophagy, migration, proliferation, survival and senescence. Indeed, it is a critical theme that BMSCs exert beneficial effects against various diseases through paracrine factor production rather than differentiation. For example, BMSCs exhibit their immunomodulatory properties in the experimental moles of heart disease and stroke by secreting transforming growth factor beta 1 (TGF-β1) [10–12]. Moreover, TGF-β proteins have been explored in recent years due to the promising potential they hold in the regulation of cell autophagy [13, 14]. However, whether BMSCs contribute to improving epidermal cell autophagy dysfunction to promote diabetic wound healing through their paracrine effects or the role of BMSC-derived TGF-β1 during this process has not been reported. Thus, there is a strong need for insight into the potential pro-autophagy effect of BMSCs on diabetic wound healing, especially the link between BMSC-derived TGF-β1 and epidermal cell autophagy.

Hypoxia is not only a vital component of the BMSC microenvironment in bone marrow but also a typical pathological phenomenon of diabetic wounds that surround the transplanted BMSCs. Our previous studies have shown that cells on the wound site are normally deprived of O_2_ [15], which is mainly attributed to enhanced oxygen consumption and insufficient blood supply to wound tissue [16]. In addition, compelling evidence is accumulating for the critical influence of the microenvironment surrounding BMSCs on BMSC-secreted bioactive molecules, which then exhibit paracrine effects on neighboring cells [17]. Therefore, an exploration of the paracrine activity of hypoxic BMSCs can aid in improving our understanding of the therapeutic effect of BMSCs for diabetic wound repair.

In this study, we demonstrated that hypoxia enhanced TGF-β1 secretion by BMSCs, which was mediated by hypoxia-induced factor-1 alpha (HIF-1α), and BMSCs exert a positive therapeutic benefit for diabetic wound healing by restoring epidermal cell autophagy through the HIF-1α/TGF-β1/SMAD pathway.

## Methods

### Ethics statement

All animal procedures in this study were conducted in accordance with the committee guidelines of Shenzhen People’s Hospital for animal experiments, which met the NIH guidelines for the care and use of laboratory animals (NIH Pub. No. 85-23, revised 1996).

### Cell culture

hBMSCs were purchased from American Type Culture Collection (ATCC, Manassas, USA). mBMSCs were obtained from Cyagen Biosciences Company (Guangzhou, China). These cells were cultured with a Mesenchymal Stem Cell Growth Kit for Bone Marrow-derived MSCs (ATCC PCS-500-041) (supplemented with rh FGF basic: 125 pg/mL, rh IGF-1: 15 ng/mL, 7% fetal bovine serum (FBS) and L-Alanyl-L-Glutamine: 2.4 mM). Hypoxic preconditioning was induced in an oxygen control incubator (Smartor 118, Zhejiang, China) filled with 5% CO_2_ and 90–94% N_2_. The oxygen concentrations and hypoxic incubation times were indicated as shown. Cells cultured under normoxic conditions (21% O_2_) served as a control.

The human keratinocyte (HaCaT) cell line was purchased from the Cell Bank of the Chinese Academy of Sciences (Shanghai, China) (originally from American Type Culture Collection (Manassas, USA)) and was cultured in Minimum Essential Medium (MEM; Procell Life Science & Technology, PM150410) containing 10% FBS (Gibco, A3160802) and 1% penicillin and streptomycin (Gibco). HaCaT cells were treated with high glucose (HG) (25 mM glucose) and 1% O_2_ hypoxia.

### Preparation of CM

To collect CM from hypoxia-preconditioned BMSCs, 3 × 10^5^ BMSCs were seeded into 10-cm dishes and grown to 80% confluence. The culture medium was then replaced with DMEM containing 0.1% FBS, and the cells were cultured under hypoxic or normoxic conditions. Then, the media was collected.

### Western blot analysis

Cells were harvested and washed twice with ice-cold PBS and lysed with protein extraction agent (Beyotime, Beijing, China). Proteins (25–50 μg) were loaded and run on a 6–10% SDS/PAGE gel and then transferred onto PVDF membranes (Millipore, Billerica, MA). After PVDF membranes were blocked at room temperature for 1 h, the membranes were incubated with primary antibodies overnight at 4 °C. The primary antibodies used in the study included HIF-1α (1:5000, Proteintech, 20960–1-AP), TGF-β1 (1:1000, Abcam, ab215715), TGF-β2 (1:1000, Abcam, ab36495), TGF-β3 (1:1000, Abcam, ab15537), ATG5 (1:1000, HUABIO, ET1611-38), ATG7 (1:1000, HUABIO, ET1610-53), LC3B (1:1000, HUABIO, ET1701-65), BECN1 (1:1000, HUABIO, R1509-1), SQSTM1 (1:500, HUABIO, R1309-8), SMAD2 (1:1000, CST, #5339), phospho-SMAD2 (1:1000, CST, #18338), SMAD4 (1:1000, CST, #8685), mTOR (1:1000, CST, #2983), p-mTOR(ser2448) (1:1000, CST, #5536), ERK1/2 (1:1000, CST, #4695), p-ERK1/2 (1:2000, CST, #4370), β-Actin (#3700 s, 1:1000, CST), GAPDH (1:1000, CST, #5174S), and Lamin B1 (1:1000, CST, #13435S). After that, the membranes were incubated with secondary antibodies for 1 h at room temperature. The protein bands were visualized using Millipore’s enhanced chemiluminescence (ECL) system and detected using MultiImage Light Cabinet Filter Positions (Alpha Innotech, San Leandro, CA, USA). The intensity of each band was analyzed by ImageJ software (NIH, Bethesda, MD, USA).

### Immunofluorescence (IF) staining

After being treated, the cells on glass coverslips were fixed in 4% paraformaldehyde for 30 min, and mouse skin was fixed in 4% paraformaldehyde for 2 days and then resected for frozen sectioning. The samples were washed three times with PBS, blocked with 10% goat serum for 1 h, nested, incubated with primary antibodies at 4 °C overnight and washed three times with phosphate-buffered saline (PBS). Next, the samples were stained with fluorescent secondary antibodies for 1 h at 37 °C. The following primary antibodies were used: HIF-1α (1:200, Proteintech, 20960-1-AP), ATG5 (1:200, HUABIO, ET1611-38), ATG7 (1:200, HUABIO, ET1610-53), K14 (1:200, Proteintech, 60320-1-Ig), and SMAD4 (1:200, CST, #8685). Nuclei were then counterstained with DAPI (Abcam) before imaging. IF images were captured using fluorescence microscopy and confocal microscopy (Leica Microsystems, Germany).

### Cell viability assay

Cell viability was determined after 12, 24, 36, 48 and 72 h of incubation with different media at 37 °C by a Cell Titer 96® Aqueous One Solution Cell Proliferation Assay (MTS) from Promega (Madison, WI, USA), a colorimetric method for determinating the number of viable cells in proliferation assays. All experiments were repeated in triplicate.

### ELISA

CM from normoxic and hypoxic BMSCs were collected and subjected to ELISA analysis to determine the TGF-β1, TGF-β2, and TGF-β3 contents. ELISA kits from R&D Systems (Minneapolis, MN, USA) were used according to the manufacturer’s protocols. Concentrations were normalized to the total protein content.

### Ethynyl-2′-deoxyuridine (EdU) assay

Cell proliferation was also measured using EdU assay kit EdU assay kit (RiboBio, C10310-1) according to the manufacturer’s instructions. Briefly, cells were seeded into 24-well plates at a density of 5.0 × 10^4^ cells/well and cultured for 24 h before the administration of Edu (50 mM). Then, Apollo and DNA stains were added. Finally, proliferation images were acquired and analyzed by fluorescence microscopy (Leica Microsystems, Germany).

### Scratch assay

We utilized a scratch assay to measure HaCaT cell migration. HaCaT cells were plated on 6-well plates. Cell monolayers were scratched with 200 µl plastic pipets. At 24 h after wounding, cells that migrated into the cell-free area were monitored with an inverted light microscope (Olympus, Japan). Cell migration was assessed by the residual wound rate: (residual scratch width / original scratch width) × 100%.

### siRNA transfection

On the day prior to transfection, cells were plated to the required cell density (at least 70% confluence). Small interfering RNAs (siRNAs) specific for ATG5, ATG7, TGF-β1, SMAD2, HIF-1α and the corresponding scrambled siRNA (siNC) were diluted in Opti-MEM (Life Technologies) and incubated for 5 min at room temperature. The diluted siRNAs were added to diluted Lipofectamine 2000 (Invitrogen, USA) and further incubated for 20 min. The complex was added according to the manufacturer's protocol.

### Quantitative real-time reverse transcription PCR (qRT-PCR)

AG RNAex Pro RNA Reagent (AG21102, Accurate Biotechnology(Hunan)Co., Ltd) was used to extract total RNA from cells. Complementary DNA (cDNA) was synthesized using the PrimeScript RT reagent kit (RR037A, Takara, Japan) and real-time PCR was performed with TB Green Premix Ex Taq (RR420A, Takara, Japan) on a StepOnePlus quantitative PCR system (Applied Biosystems, USA). Expression levels were normalized to the internal control (GAPDH) and the relative expression levels were evaluated using the comparative 2^−ΔΔCT^ method. The specific primers for ATG5, ATG7 and GAPDH were purchased from Sangon Biotech Co., Ltd. (Shanghai, China). The primer sequences are listed in Additional file 1: Fig. S1.

### Full-thickness cutaneous wound model

A total of thirty-six 8-week-old male type-2 diabetic mice (db/db) were acquired from Shanghai Slac Laboratory Animal Co., Ltd. (Shanghai, China). The full-thickness cutaneous wound model was established as previously described. Briefly, the mice were anesthetized by 5% pelltobarbitalum natricum (25 mg/kg) and Sumianxin (0.1 ml/kg), and an 8-mm diameter wound was created by a punch biopsy instrument with moderate force on the back of the mouse. Next, the middle of the outlined region of skin was sharply excised along the outline with a pair of scissors. The excised tissues were full-thickness in depth, leaving subcutaneous dorsal muscle exposed after the excision. Before being injected, mBMSCs were transfected with TGF-β1 siRNA or negative control siRNA, 2 × 10^6^ cells were suspended in 4 ml of PBS, and then the cells were intradermally injected around each wound. PBS (4 ml) was used as a control.

### Haematoxylin and eosin (H&E)

For histological analysis, the excised skin samples were fixed in 4% paraformaldehyde for 24 h, embedded in paraffin, and then stained with H&E (Sigma, Poole, UK). The stained sections were scanned for digital imaging by light microscopy (Olympus BX51, Olympus, Japan).

### Statistical analyses

All experiments were performed at least in triplicate to be eligible for the indicated statistical analysis. Statistical comparisons were then performed with GraphPad Prism 9.0 (GraphPad Software). All data are presented as the mean ± SD. The Shapiro–Wilk test was used to check whether the data were normally distributed. Unpaired t test was used to examine the difference if the data satisfied the normality requirement, otherwise, Wilcoxon test was used. Statistical significance among three or more groups was assessed by one-way analysis of variance (ANOVA), and differences between two or more groups at different time points were estimated using two-way ANOVA. The Bonferroni method was used for multiple-group comparisons after ANOVA. A two-sided P value of less than 0.05 was considered statistical significance (*P < 0.05, **P < 0.01).

## Results

### Hypoxic conditions promoted human BMSC (hBMSC) survival and HIF-1α-mediated TGF-β secretion

**Fig. 1:**
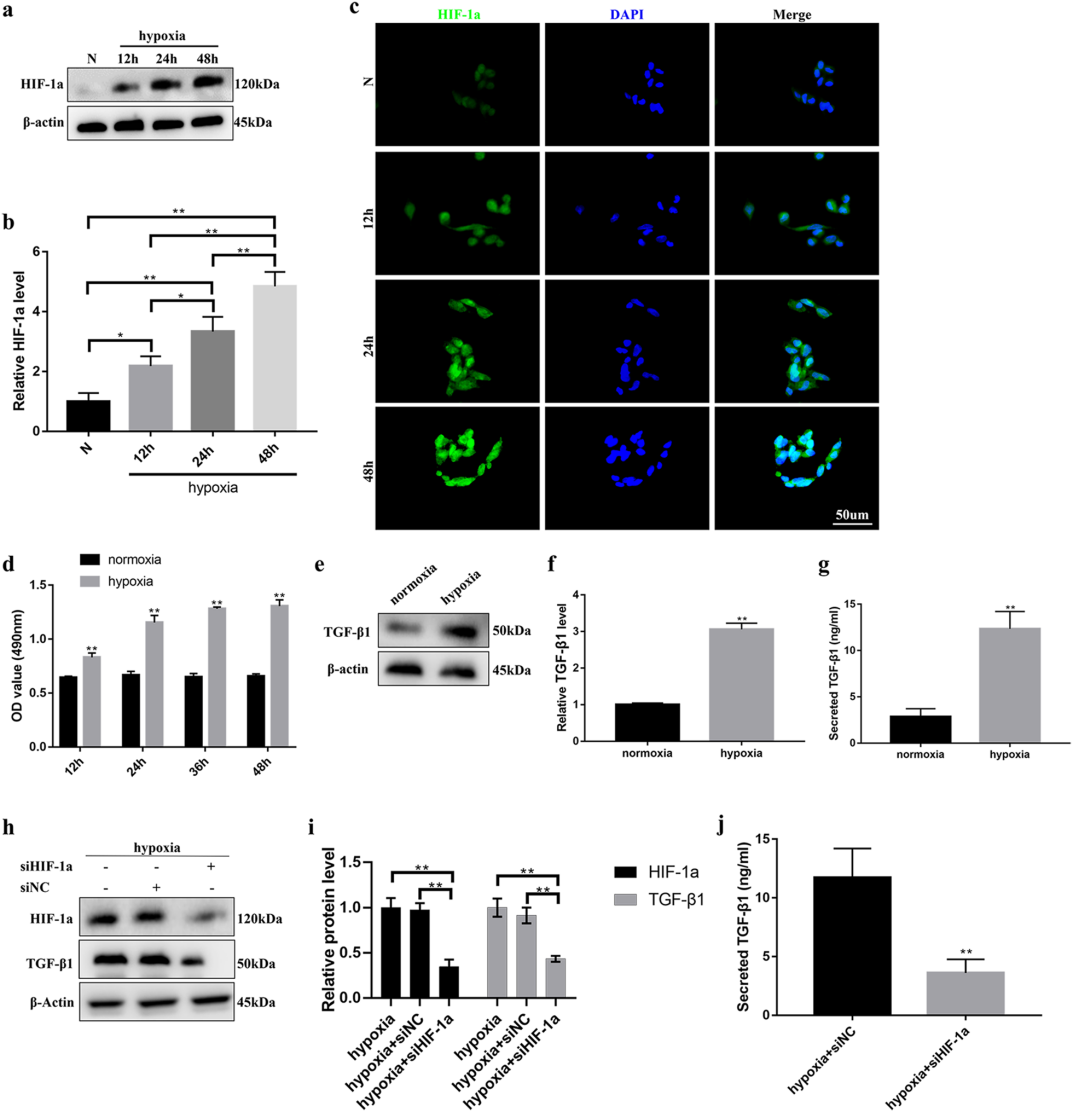
Hypoxia favors hBMSC survival and HIF-1α-mediated TGF-β1 secretion. **a** Western blotting and **b** quantitative analysis were used to analyze the expression level of HIF-1α in hBMSCs after different times (12, 24 and 48 h) of hypoxia (1% O_2_) and normoxia. **c** Representative fluorescence images of HIF-1α-stained hBMSCs under different hypoxic and normoxic conditions. Bar, 50 μm. **d** The viability of hBMSCs was determined using MTS assays after hypoxia and normoxia for different times (12, 24, 36 and 48 h). **e** Western blotting and **f** quantitative analysis were used to analyze the expression levels of TGF-β1 in hBMSCs after 48 h of hypoxic and normoxic conditions. **g** ELISA was performed to measure the protein level of secreted TGF-β1 in CM from hBMSCs subjected to 48 h of hypoxic and normoxic conditions. **h** Western blotting and **i** quantitative analysis were used to analyze the expression levels of HIF-1α and TGF-β1 in control hBMSCs and siHIF-1α hBMSCs after 48 h of hypoxia. **j** ELISA was performed to measure TGF-β1 secretion levels in the CM of control hBMSCs and siHIF-1α hBMSCs after 48 h of hypoxia treatment. Mean ± SEM. *n* = 3. *P < 0.05, **P < 0.01

To confirm the establishment of a hypoxic model, we first examined the expression of HIF-1α in hBMSCs after different times (12, 24 and 48 h) of hypoxic conditions (1% O_2_) and normoxic conditions (Fig. 1a–c). Next, the MTS assay was utilized to evaluate BMSC viability subjected to hypoxic or normoxic conditions for different times (12, 24, 36 and 48 h). As shown in Fig. 1d, the viability of BMSCs under hypoxic conditions was significantly higher than that under normoxic conditions at different periods, suggesting that hypoxic conditions favor BMSC survival. Considering that the paracrine activity of BMSCs is potentiated by hypoxia and exerts beneficial effects against tissue damage [18], we investigated whether hypoxia enhanced the expression and secretion of TGF-β1 in BMSCs. Western blot results indicated that hypoxic conditions for 48 h significantly promoted the expression of TGF-β1 in hBMSCs compared with normoxic conditions (Fig. 1e, f). We further measured and compared the levels of TGF-β1 in hypoxic hBMSC-derived conditioned medium (hyCM) vs. normoxic hBMSC-derived conditioned medium (noCM) by ELISA. As shown in Fig. 1g, the level of TGF-β1 was significantly higher in hyCM that in noCM. Next, we explored how hypoxic conditions stimulate the expression and secretion of TGF-β1 in hBMSCs. Of note, recent studies identified HIF-1α as a key regulator of TGF-β1 expression in hypoxic cells [19, 20]. Our results also indicated that inhibiting HIF-1α expression by siRNA interference decreased TGF-β1 expression and secretion in hBMSCs subjected to hypoxia (Fig. 1h–j), suggesting that HIF-1α participated in the induction of TGF-β1 in hBMSCs in response to hypoxic conditions.

### CM from hypoxic hBMSCs induced HaCaT cell autophagy, proliferation and migration

**Fig. 2:**
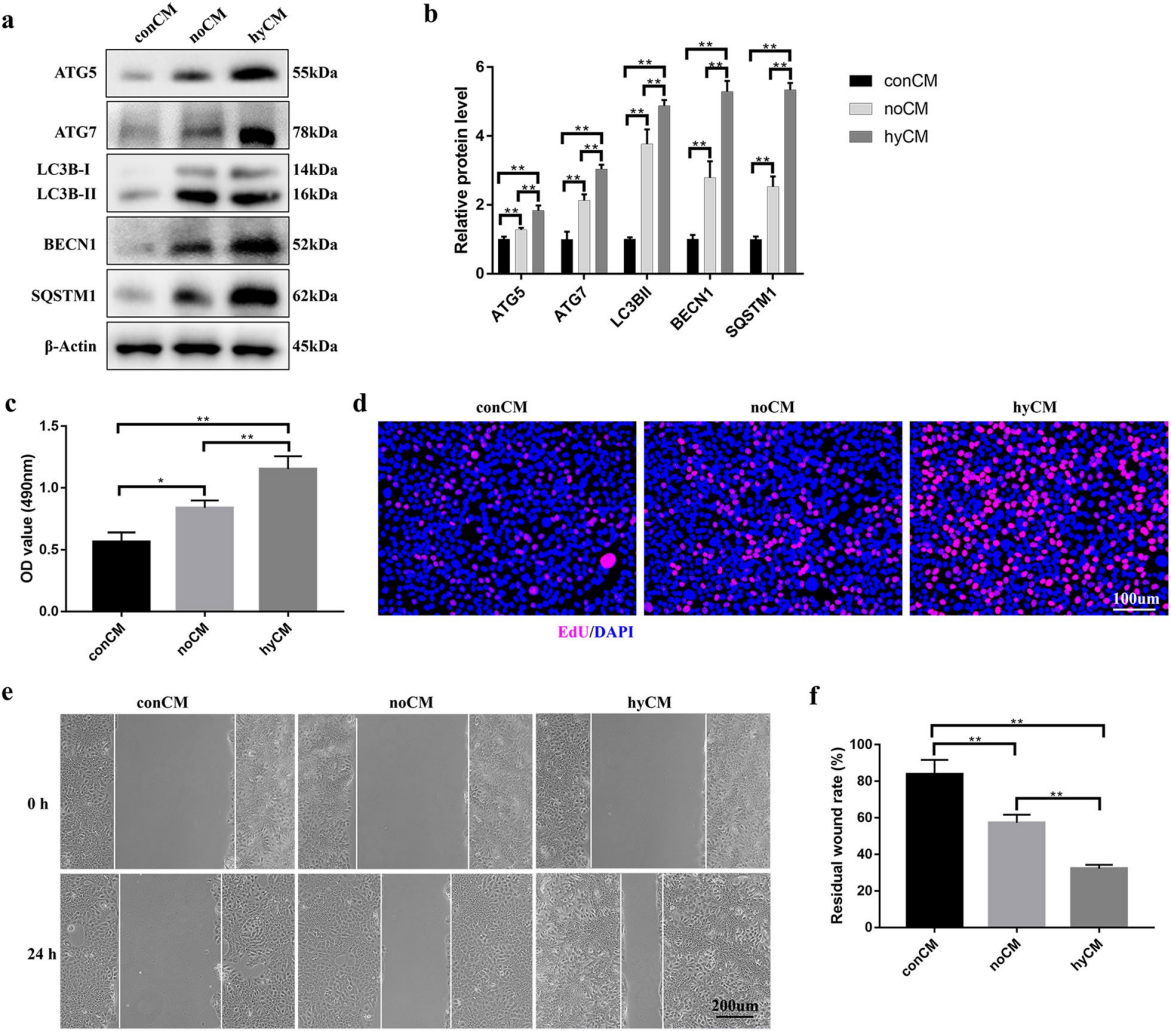
Hypoxic hBMSCs promote HaCaT cell autophagy, proliferation and migration in a paracrine manner. **a** Western blotting and **b** quantitative analysis were used to analyze the expression levels of ATG5, ATG7, LC3B-I/II, BECN1 and SQSTM1 in HaCaT cells after treatment with different conditioned media for 24 h. **c** MTS and **d** EdU assays were used to assess the proliferation of HaCaT cells after 24 h of treatment with different CM. Bar, 100. **e** Scratch assays and **f** quantitative analysis were performed to detect the migration of HaCaT cells after the cells were treated with different conditioned media for 24 h. Bar, 200 μm. Mean ± SEM. *n* = 3. *P < 0.05, **P < 0.01

To identify the therapeutic effect of hypoxic hBMSCs on epidermal cells in a paracrine manner, we incubated high glucose-treated (HG-treated) HaCaT cells under hypoxic conditions in different CMs, including hyCM and noCM, for 24 h. No hBMSC-derived CM treatment served as a control (conCM). Next, the expression of autophagy-related proteins was analyzed by Western blotting, and the results demonstrated significant increases in the autophagy-related proteins ATG5, ATG7, LC3B-I/II, BECN1 (beclin-1) and SQSTM1 (p62, the autophagy receptor) in the hyCM group compared with the noCM and conCM groups (Fig. 2a, b). Because epidermal cell autophagy enables epidermal cell proliferation and migration [4], we further evaluated the beneficial effects of hyCM on HaCaT cell proliferation and migration. The MTS assay results indicated marked upregulation of HaCaT cell viability in the hyCM group compared with the other two groups (Fig. 2c). Likewise, the results of the EdU proliferation assay demonstrated that the number of EdU-stained HaCaT cells was higher in both the hyCM and noCM groups than in the conCM group (Fig. 2d). Furthermore, hyCM treatment increased the number of EdU-stained cells (Fig. 2d). In addition, a scratch assay revealed that hyCM induced more HaCaT cell migration than that in the noCM and conCM groups (Fig. 2e, f). These results confirmed that hypoxic hBMSCs support HaCaT cell autophagy, proliferation and migration in a paracrine manner.

### The proliferative and migratory effects of hypoxic hBMSCs on HaCaT cells could be blocked by suppressing autophagy

**Fig. 3:**
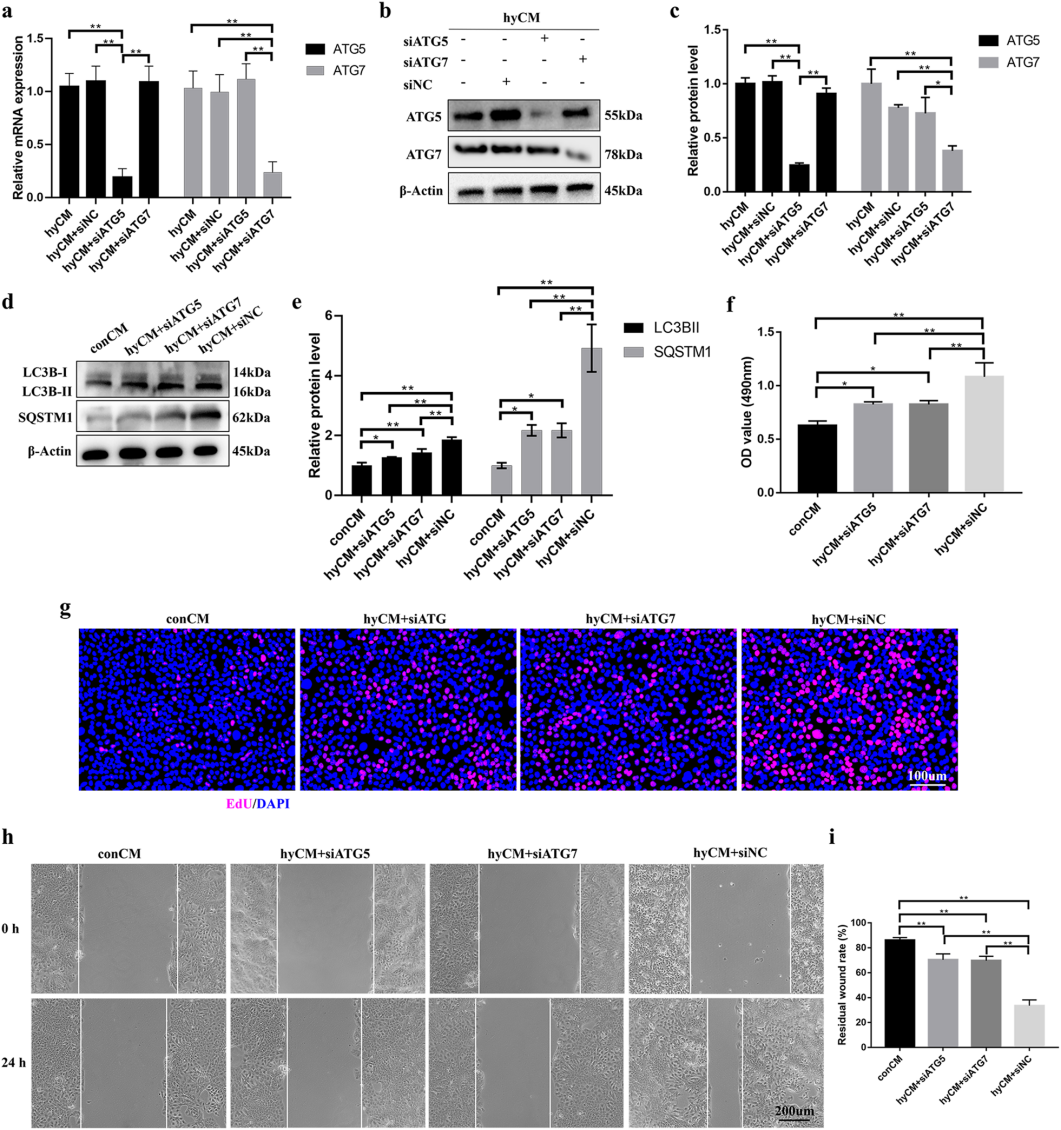
The autophagic effect of hypoxic hBMSCs promotes HaCaT cell proliferation and migration. **a** qRT-PCR and **b**, **c** Western blotting was used to measure the mRNA and protein levels of ATG5 and ATG7 to evaluate the efficiency of siATG5 and siATG7 in HaCaT cells at 24 h after hyCM administration. **d** Western blotting and **e** quantitative analysis were used to analyze the expression levels of LC3B-I/II and SQSTM1 in control HaCaT cells and siATG5 and siATG7 HaCaT cells after the cells were treated with different conditioned media for 24 h. **f** MTS and **g** EdU assays were used to assess the proliferation of these HaCaT cells after 24 h of treatment with different CM. Bar, 100. **h** Scratch assays and **i** quantitative analysis were performed to detect the migration of HaCaT cells after treatment with different conditioned media for 24 h. Bar, 200 μm. Mean ± SEM. *n* = 3. *P < 0.05, **P < 0.01

To further identify the biological meaning of the autophagic effect of hypoxic hBMSCs on HaCaT cell proliferation and migration, we first used a stable siRNA expression system targeting the essential autophagy genes ATG5 and ATG7 in HaCaT cells subjected to hyCM for 24 h. PCR and Western blotting were used to examine the transfection efficiency of siRNA in HaCaT cells (Fig. 3a–c). Moreover, LC3B-I/II and SQSTM1 degradation indicated that siATG5 and siATG7 were effective in suppressing autophagic flux (Fig. 3d, e). The results of the MTS and EdU proliferation assays showed that the proliferation of HaCaT cells exposed to hyCM was significantly attenuated due to autophagy defects (Fig. 3f, g). Then, we examined HaCaT cell migration potential during autophagy dysfunction. As shown in Fig. 3h, i, HaCaT cells with impaired autophagy exhibited delayed wound closure in the presence of hyCM, suggesting that the migratory effect of hypoxic hBMSCs was inhibited due to HaCaT cell autophagy deficiency. In summary, the effect of hypoxic hBMSCs on epidermal cell autophagy contributes to promoting HaCaT cell proliferation and migration.

### TGF-β1 was required for the effect of hypoxic hBMSCs on HaCaT cell autophagy

**Fig. 4:**
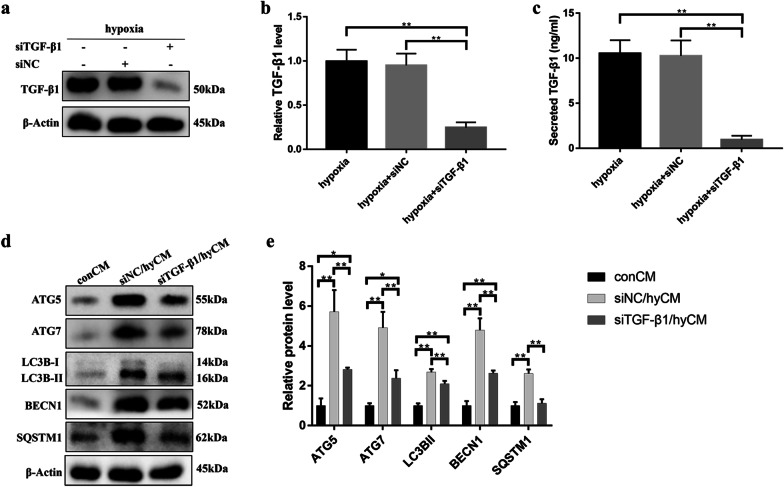
hBMSC-derived TGF-β1 contributes to the activation of HaCaT cell autophagy by hypoxic hBMSCs. **a** Western blotting and **b** quantitative analysis were used to measure the protein level of TGF-β1 to evaluate the efficiency of siTGF-β1 in hBMSCs after 24 h of hypoxia exposure. **c** ELISA was performed to measure the protein level of secreted TGF-β1 in the CM from control hBMSCs and siTGF-β1 hBMSCs after 24 h of hypoxia. **d** Western blotting and **e** quantitative analysis were used to analyze the expression levels of ATG5, ATG7, LC3B-I/II, BECN1 and SQSTM1 in HaCaT cells after 24 h of exposure to CM from control hy-hBMSCs and siTGF-β1 hy-hBMSCs. Mean ± SEM. *n* = 3. *P < 0.05, **P < 0.01

Next, we tested the hypothesis that hBMSC-derived TGF-β1 is responsible for the activation of epidermal cell autophagy by CM from hypoxic hBMSCs. We used hypoxic hBMSCs (hy-hBMSCs) transfected with TGF-β1 siRNA (hypoxia + siTGF-β1) or negative control siRNA (hypoxia + siNC). We confirmed the successful knockdown of TGF-β1 in siRNA-transfected hy-hBMSCs by analyzing TGF-β1 levels in hy-hBMSCs and CM from hy-hBMSCs (Fig. 4a–c). Further, we examined the expression of autophagy-related proteins in HaCaT cells exposed to CM. As shown in Fig. 4d, e, Western blot analysis revealed that the expression of autophagy-related proteins was lower in HaCaT cells cultured in CM from siTGF-β1/hy-hBMSCs (siTGF-β1/hyCM) than in HaCaT cells cultured in CM from siNC/hy-hBMSCs (siNC/hyCM). These results indicate the paracrine effect of TGF-β1 on hy-hBMSC-induced HaCaT cell autophagy.

### The SMAD signaling pathway functioned downstream of BMSC-derived TGF-β1 in the regulation of HaCaT cell autophagy

**Fig. 5:**
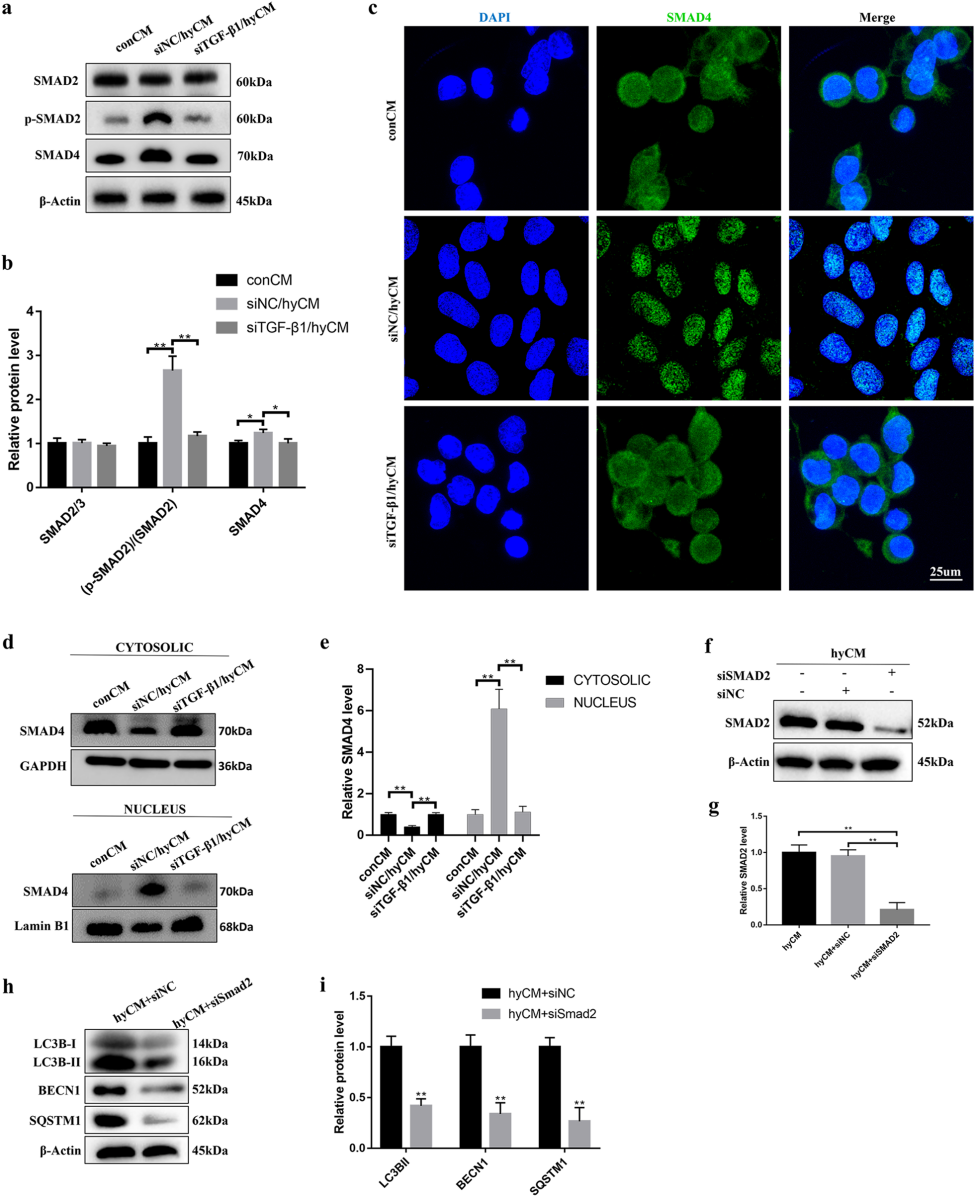
The SMAD pathway is responsible for HaCaT cell autophagy induction by hBMSC-derived TGF-β1. **a** Western blotting and **b** quantitative analysis were used to analyze the expression levesl of SMAD2, p-SMAD2 and SMAD4 in HaCaT cells subjected to CM from control hy-hBMSCs and siTGF-β1 hy-hBMSCs for 24 h. **c** Representative fluorescence images of SMAD4-stained HaCaT cells after 24 h of treatment with different CM. Bar, 20 μm. **d** Western blotting and **e** quantitative analysis were used to examine the proportion of SMAD expression in the nucleus and cytoplasm of HaCaT cells subjected to 24 h of different CM. **f** Western blotting and **g** quantitative analysis were used to measure the protein level of SMAD2 to evaluate the efficiency of siSMAD2 in HaCaT cells at 24 h after hyCM administration. **h** Western blotting and **i** quantitative analysis were used to analyze the expression levels of LC3B-I/II, BECN1 and SQSTM1 in control HaCaT cells and siSMAD2 HaCaT cells at 24 h after hyCM administration. Mean ± SEM. *n* = 3. *P < 0.05, **P < 0.01

Notably, at the molecular level, the regulation of TGF-β1 for the expression of autophagy genes is indirect and needs to be mediated by downstream signaling pathways. Since SMAD and the non-SMAD-dependent signaling pathway function downstream of TGF-β1 in regulating many fundamental aspects of cellular behavior, we investigated whether these pathways contribute to this process. As shown in Fig. 5a, b, siTGF-β1/hyCM significantly downregulated the phosphorylation level of SMAD2 and slightly changed total SMAD4 compared with the effects of siNC/hyCM. However, IF analysis showed that SMAD4 was mainly localized to the nucleus in HaCaT cells in the siNC/hyCM group compared to those in the siTGF-β1/hyCM and conCM groups (Fig. 5c). Thus, we further used Western blot analysis to examine SMAD4 expression in the nucleus and cytoplasm of HaCaT cells. Consistent with the IF data, SMAD4 was significantly upregulated in the nuclei of HaCaT cells in the siNC/hyCM group, while the nuclear expression of SMAD4 protein was notably suppressed in the other groups (Fig. 5d, e). In addition, the activities of other SMAD-independent pathways were not significantly different among the three groups (Additional file 1: Fig. S2). Next, we investigated the role of the SMAD pathway through a loss-of-function assay. After SMAD2 siRNA was transfected into HaCaT cells (Fig. 5f, g), the activation of HaCaT cell autophagy was significantly attenuated in the presence of hyCM (Fig. 5h, i). These results suggest that the SMAD signaling pathway acts downstream of hBMSC-derived TGF-β1 to regulate HaCaT cell autophagy.

### Knockdown of TGF-β1 in mouse BMSCs (mBMSCs) reduced the therapeutic effect on diabetic wounds by attenuating the autophagic potential of mBMSCs

**Fig. 6:**
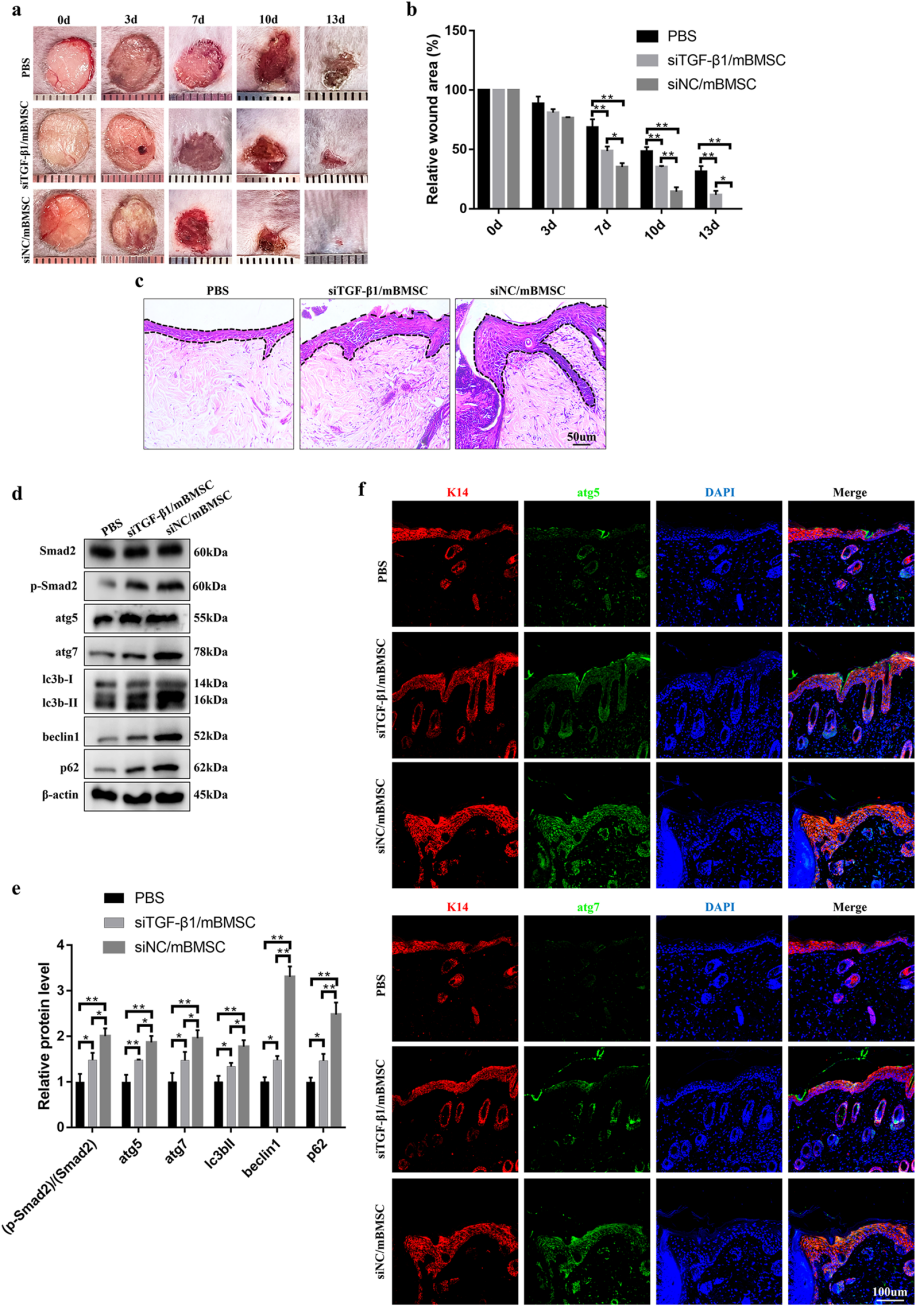
Knockdown of TGF-β1 in mBMSCs attenuates the therapeutic effects on diabetic wounds. **a** Representative pictures and **b** quantitation of the healing time of control mBMSC-treated and siTGF-β1 mBMSC-treated and untreated diabetic wounds after an 8-mm primary biopsy. *n* = 6. **c** Representative H&E-stained images of the wounds on day 7. The epithelium is marked with a dotted line. Bars, 50 μm. **d** Western blotting and **e** quantitative analysis were used to analyze the expression levels of Smad2, p-Smand2, atg5, atg7, lc3b-I/II, beclin1 and p62 in the wounds on day 7 after wounding. **f** Representative images of wounded (day 7) skin stained to show the expression of atg5 or atg7 (green) and K14 (red) in the wounds (blue). Bar, 100 μm. Mean ± SEM. *n* = 3 (in addition to the quantitation of healing time). *P < 0.05, **P < 0.01

To further investigate the therapeutic effect of BMSCs on diabetic wound repair and the role of the TGF-β1/SMAD signaling pathway in this process, diabetic wounds were administered differentially treated mBMSCs on three consecutive days (days 0–2), and the quality of wound healing was further evaluated. The results showed that mBMSC treatment remarkably accelerated wound closure from day 7 to day 13, which was partly attenuated due to the knockdown of TGF-β1 in mBMSCs (Fig. 6a, b). The H&E staining results of day 7 wounds further showed that the newly healed wounds in the siNC/mBMSC group resulted from rapid re-epithelialization, which was reversed due to TGF-β1 interference in the siTGF-β1/mBMSC group (Fig. 6c). In the present study, we found that the expression of autophagy-related proteins in wound tissue was significantly induced in the siNC/mBMSC group at 7 days postwounding, accompanied by the upregulation of Smad2 phosphorylation levels compared with those in the other two groups (Fig. 6d, e). In addition, we performed double immunostaining for keratin 14 (K14) (a keratinocyte marker) and atg5 or atg7 to identify keratinocyte-specific autophagy at 7 days postwounding. A small number of cells that were positive for both K14 and atg5 or atg7 were detected in the siTGF-β1/mBMSC group, and larger numbers of cells were observed in the siNC/mBMSC group (Fig. 6f). These data suggest that BMSC-based treatment could serve as an effective agent for diabetic wound repair through the activation of epidermal autophagy, which is attributed to the TGF-β1/SMAD pathway.

## Discussion

**Fig. 7:**
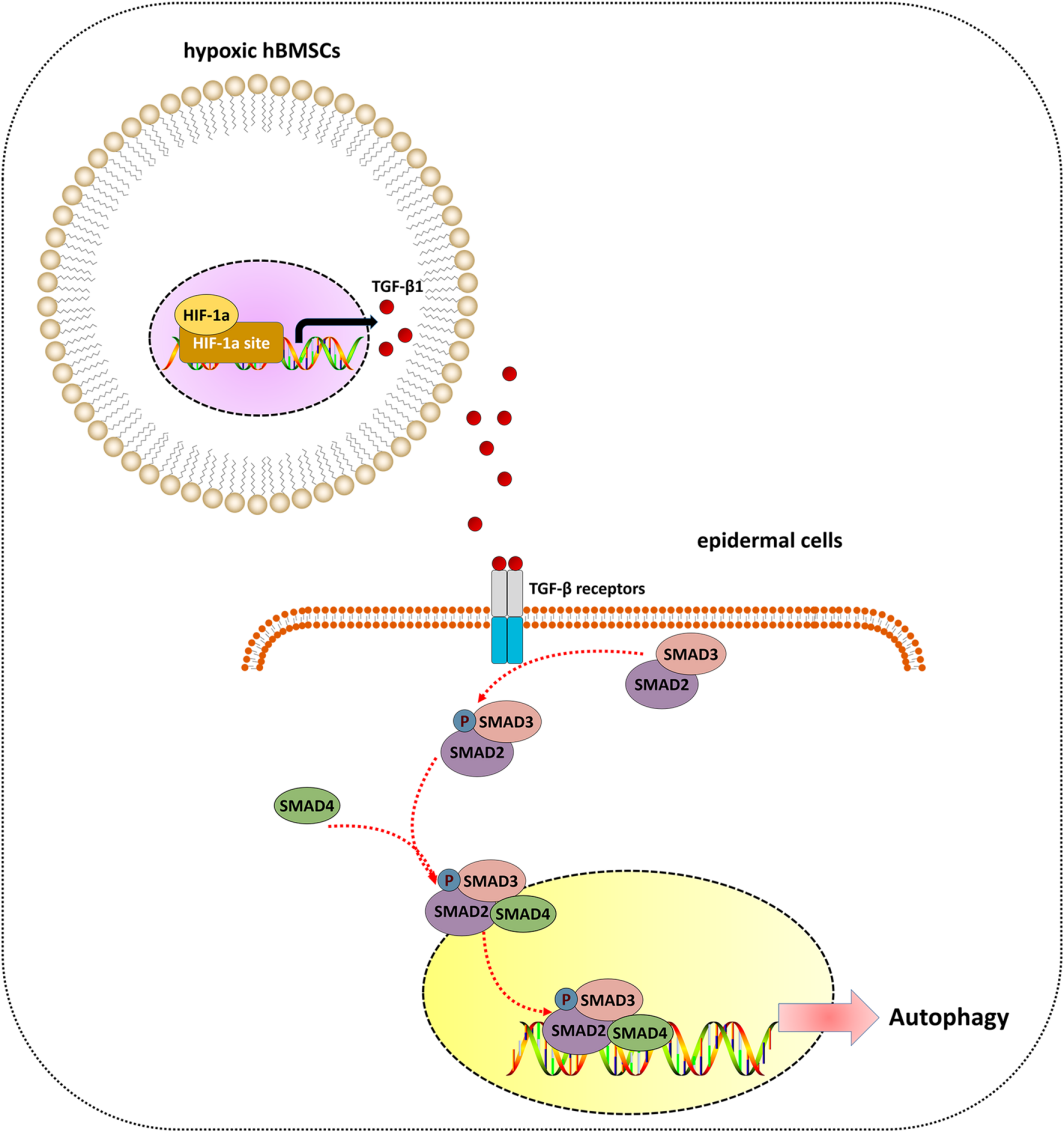
Schematic of epidermal cell autophagy activation by hypoxic hBMSCs: the primary event in this process is HIF-1α expression in hBMSCs in response to hypoxic conditions, which in turn drives TGF-β1 expression and secretion in hypoxic hBMSCs. Then, TGF-β1 from hBMSCs binds to the membrane-associated TGF-β receptors on epidermal cells and subsequently stimulates SMAD2/SMAD3 through direct phosphorylation, which further forms a trimer with SMAD4, and the complex is transferred to the epidermal cell nucleus. Activation of the SMAD pathway, in turn, induces autophagy in HaCaT cells

Since the exploration of MSC-based therapy decades ago [21], the biological activity and regenerative medicine of BMSCs have been focal topics in the broad fields of diabetic wound repair. Given the broad biological functions of BMSCs, investigating the pleiotropic effects of BMSCs and their downstream regulatory mechanisms will help to better understand BMSC-based therapeutics for diabetic wounds. In this report, we present data that revealed a previously unrecognized function of hypoxic BMSCs in the modulation of epidermal cell autophagy. First, the analysis found that TGF-β1 secretion by hypoxic BMSCs was significantly increased and that the process was mediated by HIF-1α. Furthermore, BMSC-derived TGF-β1 was responsible for activating epidermal cell autophagy, which subsequently contributed to potentiating epidermal cell proliferation and migration. Then, it was demonstrated that the SMAD-dependent signaling pathway functions downstream of BMSC-derived TGF-β1 in the regulation of epidermal cell autophagy. In vivo, BMSCs provided beneficial effects for diabetic wound healing, displaying augmentation of epidermal autophagy and rapid re-epithelialization, while downregulation of TGF-β1 in BMSCs resulted in inhibition of SMAD-dependent signaling, autophagy limitation of epidermal cells and delayed wound healing. Collectively, this study revealed a novel function of hypoxic BMSCs in the modulation of epidermal cell autophagy via the HIF-1α/TGF-β1/SMAD signaling pathway (Fig. 7), indicating that BMSC-based treatment by restoring epidermal cell autophagy could be an attractive therapeutic strategy for diabetic wounds.

During the exploration of the therapeutic effect and molecular mechanisms of BMSCs, these cells are usually exposed to normoxia (21% O_2_) under in vitro culture conditions, which is very different from the oxygen concentrations found in the body. In fact, a large proportion of BMSCs are present in a hypoxic environment (1–8% O_2_) in the bone marrow [22]. Moreover, BMSCs transplanted into diabetic wound sites inevitably faced diminished oxygen availability due to vasculopathies such as atherosclerosis and impaired vascular structure, and heightened oxygen consumption. In addition, some studies exploring the effect of locally administered BMSCs on injured tissues found that hypoxic conditions could enhance cell survival and inhibit extensive cell apoptosis, which in turn potentiated the therapeutic effect of BMSCs [23]. Most importantly, hypoxic conditions have been shown to intensify the paracrine ability of BMSCs [24, 25]. These findings led us to hypothesize that hypoxic BMSCs could secrete more TGF-β1, thereby exerting a more robust therapeutic effect than normoxic BMSCs. In this study, we found that hypoxic conditions significantly intensified TGF-β1 expression and secretion in BMSCs. Furthermore, the data demonstrated that hypoxia favors BMSCs survival, which is consistent with previous reports that the proliferation ability of BMSCs subjected to hypoxic conditions was enhanced, accompanied by the upregulation of the expression of cell cycle regulators, such as p21, p27, p53 and p-Rb [26]. Notably, hypoxia-induced TGF-β1 expression and secretion were accompanied by increased HIF-1α expression in BMSCs. Indeed, HIF-1α was not only shown to serve as a hypoxia sensor but has also been reported to be a crucial modulator of oxygen homeostasis by modulating the transcription of genes encoding proteins [27]. It has been reported that HIF-1α can bind to the hypoxia regulatory element of the TGF-β1 promoter, which is located between bp –1030 and –666 in front of the TGF-β1 promoter region, and stimulate TGF-β1 production in BMSCs [28]. Our results further indicated that knockdown of HIF-1α by siRNAs attenuated TGF-β1 expression and secretion in BMSCs subjected to hypoxia, suggesting that hypoxia-induced TGF-β1 in BMSCs is tightly connected with HIF-1α expression.

At the cellular level, we found that CM from hypoxic BMSCs effectively induced not only epidermal cell autophagy but also epidermal cell migration and proliferation. Autophagy is a crucial eukaryotic catabolic system that plays a pivotal role in regulating cellular balance and physiology. Thus, we further explored the biological role of BMSC-evoked autophagy in the induction of epidermal cell migration and proliferation. We found that epidermal cell functions, including proliferation and migration, which were potentiated by CM from hypoxic BMSCs, were compromised due to autophagy deficiency in epidermal cells. This effect could be due to the following reasons: (a) autophagy provides nutrient and energy support for cells by degrading aggregated proteins, ribosomes and even damaged organelles, and autophagy inhibition leads to insufficient nutrient and energy supply and thus metabolic stress [29], thereby resulting in the inhibition of epidermal cell proliferation and migration; (b) autophagy adapts cells to oxidative damage through the maintenance of ROS at low physiological levels [30], but autophagy deficiency impairs cell viability due to uncontrolled ROS formation [31]; and (c) the autophagy pathway in cells is critical for regulating DNA damage repair [32, 33]; thus, the dysregulation of autophagy results in programmed cell death [34]. Therefore, the present findings indicated that the effect of hypoxic BMSCs on the induction of epidermal cell proliferation and migration is autophagy dependent, which not only emphasizes the value of the pro-autophagy effect of BMSCs on epidermal cells but also provides further research directions for exploring the molecular mechanisms by which autophagy influences epidermal cell proliferation and migration based on BMSC treatment.

TGF-β1, as an evolutionarily conserved secreted protein, can be read by neighboring cells and in turn activate intracellular signals to regulate a wide range of cellular processes [35]. Although the role of TGF-β1 as a key mediator of cell autophagy has been reported, there are conflicting studies about the impact of TGF-β1 in the regulation of cell autophagy. During the pathogenesis of liver fibrosis, Ning Lin et al. reported that TGF-β1 treatment ameliorates experimental hepatic fibrosis by inhibiting hepatic stellate cell (HSC) autophagy [36]. However, the effect of TGF-β1-driven autophagy has also been shown in renal epithelial cells [37] and lung epithelial cells [38]. Consistent with the results in these cells, we uncovered that BMSC-derived TGF-β1 was responsible for the activation of epidermal cell autophagy in our in vitro and in vivo system through loss-of-function assays. A distinct cellular context could be utilized to explain the contradictory observations. In HSCs, TGF-β1 treatment obstructs autophagic flux by upregulating mTOR signaling [39], which is recognized as a key negative pathway of autophagy [40]. However, TGF-β1 from BMSCs did not affect the phosphorylation of mTOR in our in vitro study (Additional file 1: Fig. S2). The broad biological effect of TGF-β1 depends on SMAD and non-SMAD mechanisms [41]. In the previous studies, ERK signaling was identified to be responsible for the pro-autophagy potential of TGF-β1 in ovarian carcinoma and SMAD4-negative pancreatic ductal adenocarcinoma [42, 43], which was not observed in our study (Additional file 1: Fig. S2). Instead, we found that CM from hypoxic BMSCs increased SMAD2 phosphorylation levels and SMAD4 localization in the nuclei of epidermal cells, suggesting SMAD pathway activation in epidermal cells. In TGF-β1/SMAD signaling, the binding of TGF-β1 to its receptor complex activates SMAD2/SMAD3 through direct phosphorylation and further forms a trimer with SMAD4 that subsequently translocates into the nucleus to facilitate gene transcription [44]. Regarding the essential role of the TGF-β1/SMAD signaling pathway in the modulation of cellular behavior, we hypothesized that SMADs are the pivotal downstream mediators of epidermal cell autophagy activation in response to hypoxic BMSC-derived CM. The present study further showed that SMAD2 inhibition reversed the autophagy induction of epidermal cells treated with hypoxic BMSC-derived CM, implying that epidermal cell autophagy induction by BMSC-derived TGF-β1 relies on SMAD-dependent mechanisms. In addition to totally different genetic contexts, another possible explanation is that TGF-β1-induced autophagic flux through SMAD or non-SMAD pathways based on signaling kinetics. As described in previous papers, EGF-induced ERK activation occurs within 10 min in some cells, e.g., mast cells, while a similar effect requires several hours in other cells, e.g., pancreatic acinar cells [45, 46]. Taken together, his study sheds new light on the rich repertoire of TGF-β1/SMAD signaling involved in the pro-autophagy effect of BMSCs in epidermal cells.

Wound repair is a complex and dynamic sequence that involves three major phases: inflammation, re-epithelialization, and resolution. It has become evident that various cells and the associated extracellular matrix orchestrate this process. Indeed, the dynamic influential nature of autophagy not only determines the proliferation and migration of epidermal cells in the wound bed but also directs the survival and fate of other cells as well as the transitions through the various phases of wound healing [47]. Previous work has shown that epidermal stem cells and progenitor cells maintain their self-renewal capability through autophagy [48]. Furthermore, autophagy is thought to be linked to the first and third phases of wound repair by triggering M1 macrophages to remove all damaged ECM debris [49] and the phenotypic shifts of fibroblasts [50]. Thus, more work needs to be performed exploring the relationship between the pro-autophagy effect of BMSCs and other cell types in the context of wound healing, which may provide added insight into the influence of BMSCs on diabetic wound repair. For stem cell-based therapy, in addition to BMSCs as an alternative to repair and regenerate tissues, adipose-derived mesenchymal stem cells (AD-MSCs) can serve as a stem cell source for many tissue engineering applications, including wound healing [3, 51], scars [52], skin photoaging [53, 54], and COVID-19 treatment [55–57]. However, whether the paracrine mechanism of AD-MSCs is involved in regulating cell autophagy during wound repair is still unclear. Thus, future studies should determine the autophagic potential of AD-MSCs in diabetic wound healing.

## Conclusions

In conclusion, this study revealed a previously unidentified role of hypoxic BMSCs in regulating epidermal cell autophagy and highlighted the relationship between epidermal cell autophagy and the restoration of cell proliferation and migration as well as diabetic wound healing. These data further present a novel mechanistic view that hypoxic BMSCs support epidermal cell autophagy by the HIF-1α/TGF-β1/SMAD pathway, which simultaneously identifies BMSC-based treatment as an attractive therapeutic strategy for the clinical intervention of diabetic wounds.

## Supplementary Information

**Additional file 1: Figure S1**. The sequences of primers were designed and used for qRT-PCR. **Figure S2**. TGF-β1 from BMSCs did not affect the activities of mTOR and ERK signaling pathways in HaCaT cells. (a) Western blotting and (b) quantitative analysis were used to analyze the expression levels of mTOR, p- mTOR, ERK1/2 and p-ERK1/2 in HaCaT cells subjected to CM from control hy-hBMSCs and siTGF-β1 hy-hBMSCs for 24 h. Mean ± SEM. *n* = 3. *P < 0.05, **P < 0.01.

## Data Availability

All data generated or analyzed during this study are included in this article.

## Abbreviations

AD-MSCs: Adipose-derived mesenchymal stem cells
BECN1: Beclin 1
BMSCs: Bone marrow mesenchymal stem cells
CM: Conditioned medium
conCM: No hBMSC-derived CM treatment served as a control
EdU: 5 Ethynyl-2′-deoxyuridine
hBMSC: Human BMSC
HG-treated: High glucose-treated
hyCM: Hypoxia hBMSC-derived CM
H*&*E: Haematoxylin and eosin
IF: Immunofluorescence
noCM: Normoxia hBMSC-derived CM
qRT-PCR: Quantitative real-time reverse transcription PCR
siNC: Negative control
siRNA: Small interfering RNA
SQSTM1/p62: Sequestosome 1
TGF-β: Transforming growth factor beta
