# Supplementary material for: Bone marrow mesenchymal stem cells facilitate diabetic wound healing through the restoration of epidermal cell autophagy via the HIF-1α/TGF-β1/SMAD pathway
Source: Stem Cell Res Ther. 2022 Jul 15;13:314. doi: 10.1186/s13287-022-02996-9 (PMC9284495; doi:10.1186/s13287-022-02996-9)
Supplement: Supplementary file 1 — Additional file 1: Figure S1. The sequences of primers were designed and used for qRT-PCR. Figure S2. TGF-β1 from BMSCs did not affect the activities of mTOR and ERK signaling pathways in HaCaT cells. (a) Western blotting and (b) quantitative analysis were used to analyze the expression levels of mTOR, p- mTOR, ERK1/2 and p-ERK1/2 in HaCaT cells subjected to CM from control hy-hBMSCs and siTGF-β1 hy-hBMSCs for 24 h. Mean ± SEM. n = 3. *P < 0.05, **P < 0.01. [file 13287_2022_2996_MOESM1_ESM.docx]

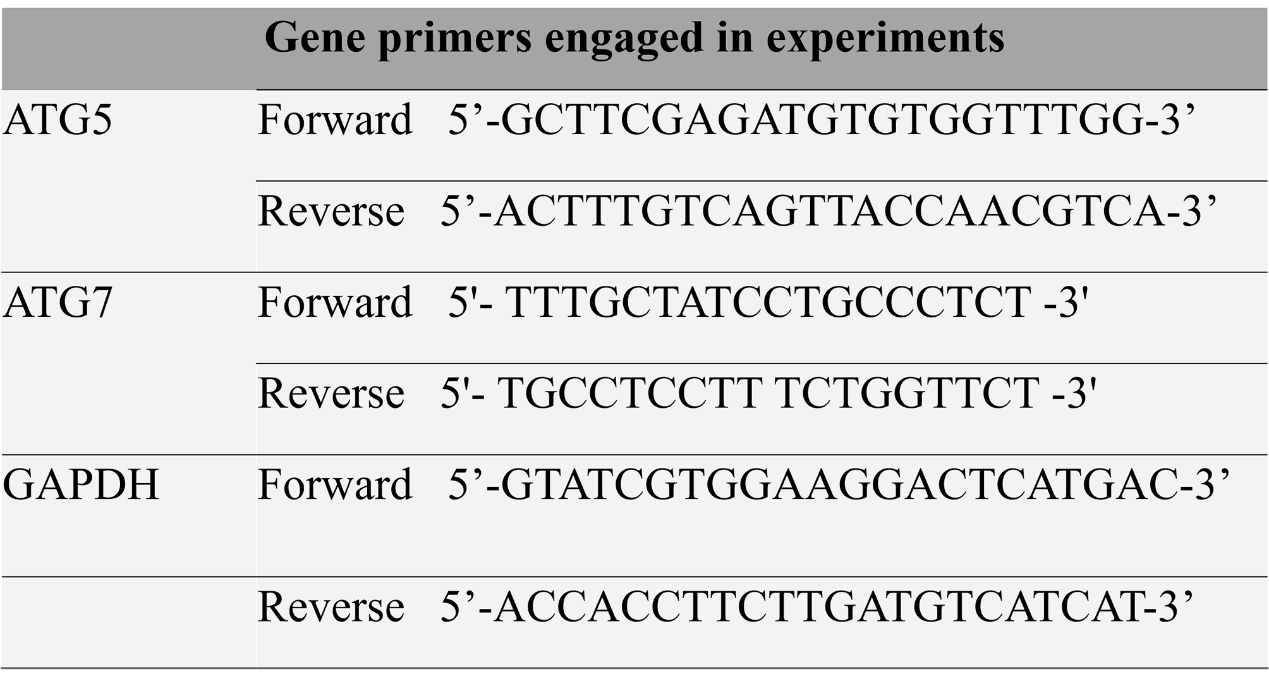
 **Figure S1. The sequences of primers were designed and used for qRT-PCR.**


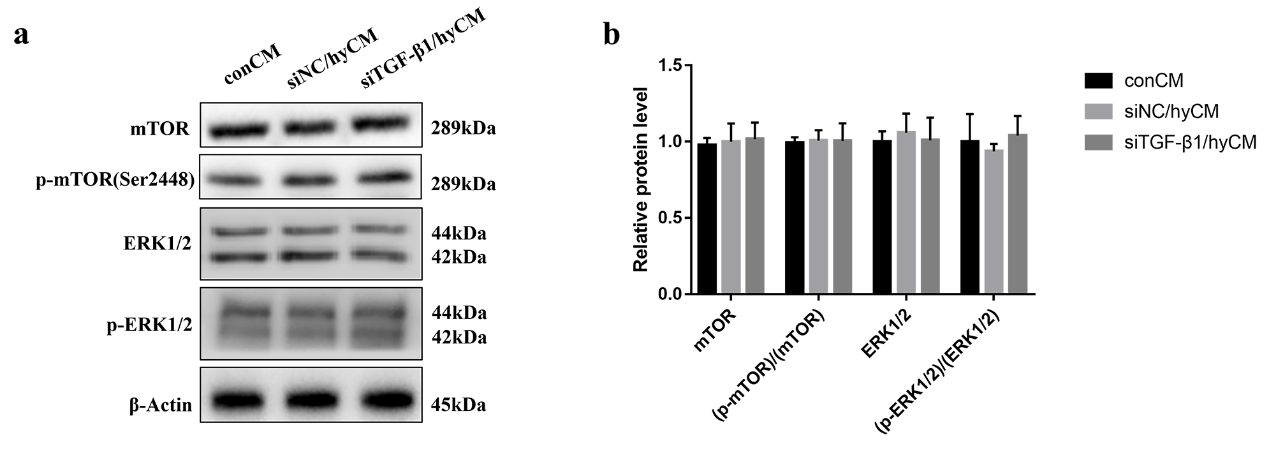


**Figure S2. TGF-β1 from BMSCs did not affect the activities of mTOR and ERK signaling pathways in HaCaT cells. (a)** Western blotting and **(b)** quantitative analysis were used to analyse the expression levesl of mTOR, p- mTOR, ERK1/2 and p-ERK1/2 in HaCaT cells subjected to CM from control hy-hBMSCs and siTGF-β1 hy-hBMSCs for 24 h. Mean ± SEM. n = 3. *P < 0.05, **P < 0.01.
